# Purification of 3, 4-dihydroxyphenylethyl alcohol glycoside from *Sargentodoxa cuneata* (Oliv.) Rehd. et Wils. and its protective effects against DSS-induced colitis

**DOI:** 10.1038/s41598-019-38926-8

**Published:** 2019-03-01

**Authors:** Dihua Li, Yuzhen Zhuo, Qi Zhang, Lanqiu Zhang, Shukun Zhang, Yuanshan Lv, Caixia Li, Lihua Cui, Xin Guan, Lei Yang, Ximo Wang

**Affiliations:** 1 Tianjin Institute of Acute Abdominal Diseases of Integrated Traditional Chinese and Western Medicine, Tianjin, 300100 China; 2 Department of Surgery, Tianjin Nankai Hospital, Tianjin, 300100 China

## Abstract

*Sargentodoxa cuneata* is a tropical plant used in traditional Chinese medicine to treat intestinal inflammation. In this study, 3, 4-dihydroxyphenylethyl alcohol glycoside (DAG) was purified from the stem of *S. cuneata* using macroporous resins and its bioactivity was also investigated. The adsorption/desorption of DAG on macroporous resins was investigated systematically. HPD300 resin was selected as the most suitable medium for DAG purification. Further dynamic absorption/desorption experiments on the HPD300 column were conducted to obtain the optimal parameters. To obtain more than 95% DAG, a second stage procedure was developed to purify the DAG using SiliaSphere C18 with 8% v/v acetonitrile through elution at low pressure. Further investigation showed that DAG pretreatment significantly reversed the shortening of colon length, the increase in the disease activity index (DAI) scores and histological damage in the colon. Moreover, DAG greatly increased SOD and GPx activities, significantly decreased MPO and MDA activities and reduced the levels of pro-inflammatory cytokines in the colon. Free radical scavenging activities of DAG were assessed using DPPH, with an IC50 value of 17.03 ug/mL. Additionally, DAG suppressed ROS and proinflammatory cytokine production in LPS-stimulated RAW 264.7 macrophages by suppressing activation of the ERK1/2 and NF-κB pathways. The results were indicative of the antioxidant and anti-inflammatory properties of DAG. When viewed together, these findings indicated that DAG can be used to expand future pharmacological research and to potentially treat colitis.

## Introduction

Phenylethanoid glycosides (PhGs) widely exist in medicinal plants, especially those used in traditional Chinese medicine (TCM). PhGs have been shown to possess outstanding pharmacological properties, such as anti-endotoxin^1^, antioxidant^2^, anti-inflammatory^3^, antivirus^4^, and antitumor^5^ effects that combat diverse diseases. Recently, research interest in PhGs has been growing. More than 100 new PhGs have been detected, isolated and identified in different plants^6^. The phenylethanoid glycoside 3, 4-dihydroxyphenylethyl alcohol glycoside (DAG) is found in many medicinal plants. However, the pharmacological effects of DAG have not been investigated. We determined through HPLC that DAG is one of the active ingredients in *Sargentodoxa cuneata* (Oliv.) Rehd. et Wils. *S. cuneata* has been used to treat ulcers in clinical studies^7^. Therefore, intensive pharmacological study of DAG is necessary for drug discovery. PhGs were reported to not only be absorbed by the lower intestine but also to be transformed by intestinal bacteria^8,9^. In this study, we investigated the antioxidant and anti-inflammatory activities of DAG in DSS-induced colitis.

DAG exists in various medicinal plants, but its concentration in different plants may vary considerably. According to reports in scientific literature, the content of DAG in the stem of *S. cuneata* can reach 10.36 mg/g^7^. Reports on the isolation and purification methods of DAG have been limited. Chen *et al*. reported on the preparation of HPLC and silica gel column chromatography for DAG^10^. However, there are several disadvantages to using this method. It is time-consuming, generates a large amount of organic solvent waste, has a poor yield and recovery ability and its use is confined to large-scale industrial production. Macroporous resins are one kind of durable hydrophilic polymer with the advantages of a high adsorption capacity, good selectivity, good stability, fast adsorption and desorption under mild conditions, ease of regeneration and cost savings. Several studies have been conducted on PhGs regarding their isolation and purification through the use of macroporous resins^11,12^. We have previously reported that macroporous resins are used as a medium by which to enrich and separate liriodendrin from the stem of *S. cuneata*^13^. In this study, a method had been established for the preparative purification of DAG from the stem of *S. cuneata* by combining macroporous resins with C18 chromatography.

## Results

### Resin screening

Nine macroporous resins with different properties were tested at 25 °C. As a result (Table 1), the polar resin had a lower adsorption capacity than other resins, and the rates of adsorption were different between different resins with the same non-polarity. The adsorption capacity of middle-polar resins was higher than AB-8 and X-5 of non-polar resins. However, the desorption rate of nine macroporous resins was not noticeably different. This may be due to the special characteristics of DAG, which is an amphipathic molecule. Besides polarity, adsorption/desorption capacity was related to the average pore diameter and the surface area of the resin. Considering the performance of adsorption and desorption, HPD100 and HPD300 resins were selected for further testing.

**Table 1:** Adsorption capacity, adsorption and desorption ratios of DAG on different resins at 25 °C.

Resin	Adsorption capacity^a^ (mg/g)	Adsorption ratios^a^ (%)	Desorption ratio^a^ (%)
AB-8	11.474 ± 0.371	61.945 ± 2.005	67.426 ± 5.938
X-5	11.317 ± 0.277	61.096 ± 1.498	70.518 ± 0.134
HPD100	15.964 ± 0.200	86.185 ± 1.077	78.481 ± 2.258
HPD300	16.669 ± 0.168	89.988 ± 0.909	78.189 ± 0.301
HPD5000	15.007 ± 0.121	81.019 ± 0.652	69.758 ± 2.169
HPD450	12.811 ± 0.460	69.164 ± 2.484	69.535 ± 0.512
HPD750	11.991 ± 0.434	64.734 ± 2.344	66.264 ± 1.003
DM130	12.547 ± 0.427	67.740 ± 2.307	68.487 ± 4.949
NKA-9	9.221 ± 0.515	49.781 ± 2.781	66.055 ± 2.719

^a^Values are means ± SD (n = 3).

### Adsorption kinetics

**Figure 1:**
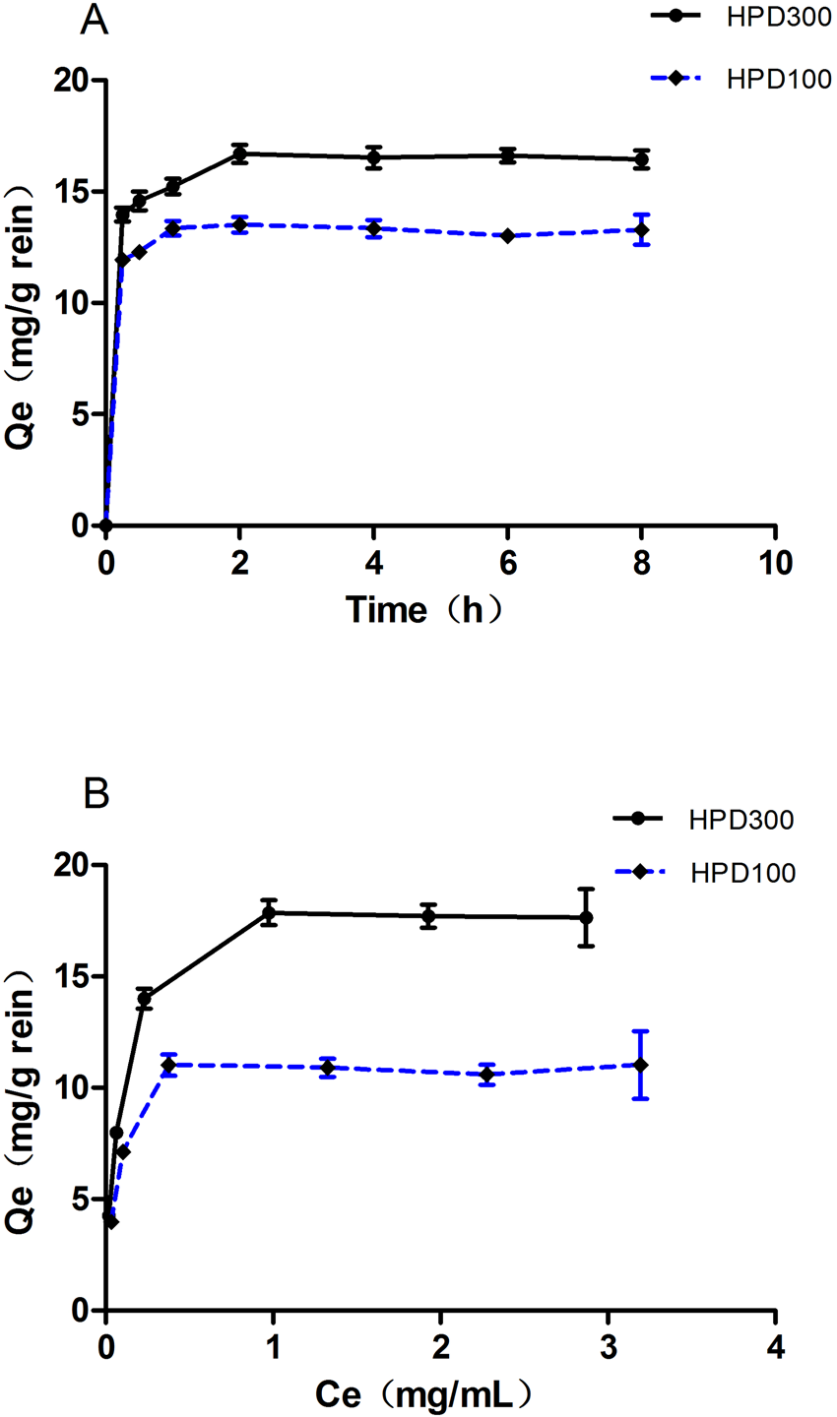
Adsorption isotherms curves (**A**) and adsorption kinetics curves (**B**) for DAG on HPD100 and HPD300 resins at 25 °C.

Adsorption kinetics on HPD100 and HPD300 resins were tested at 25 °C. Adsorption kinetic curves are shown in Fig. 1A. The adsorption rates toward DAG increased rapidly in the first 0.25 h and then continued to increase for about 1.5 h. An asymptotic curve was reached at 2 h, which indicated that the two resins were of the fast adsorption resin type^14^. The parameters of three kinetic models were calculated (Table 2). A pseudo-second-order kinetic model performed better in describing the adsorption process on HPD300 and HPD100 resins with a calculated correlation coefficient (*R*^2^ ≥ 0.999). This suggested that the adsorption process was controlled by a chemical adsorption mechanism through the sharing or exchange of electrons between the adsorbate and the adsorbent^15^. The rate constant (*k*_2_) of HPD300 was less than that of HPD100.

**Table 2:** Kinetic parameters of DAG on HPD100 and HPD300 resins at 25 °C.

Resin	HPD100	HPD300
**Pseudo-first-order**		
Equation	ln (13.1771 − *Q*_*t*_) = 2.5785 − 0.1490*t*	ln (16.1348 − *Q*_*t*_) = 2.7810 − 0.1230*t*
*R* ^2^	0.9939	0.9839
*k*_1_ (min^−1^)	0.1490	0.1230
*Q*_*e*_ (mg/g)	13.1771	16.1348
**Pseudo-second-order**		
Equation	*t*/*Q*_*t*_ = 0.0756_*t*_ + 0.0270	*t*/*Q*_*t*_ = 0.0602_*t*_ + 0.1305
*R* ^2^	0.9997	0.9998
*k*_2_ (*g*/*mg* · min)	0.2117	0.0278
*Q*_*e*_ (mg/g)	13.2275	16.6113
**Intra-particle diffusion**		
Equation	*Q*_*t*_ = 0.3675_*t*_^0.5^ + 7.4572	*Q*_*t*_ = 0.4947_*t*_^0.5^ + 8.5301
*R* ^2^	0.3751	0.4532
*k*_*i*_ (*mg*/*g* · min^0.5^)	0.3675	0.4947
*C* (mg/g)	7.4572	8.5301

### Adsorption isotherms

Equilibrium adsorption isotherms were studied for DAG on HPD100 and HPD300 resins at 25 °C. As shown in Fig. 1B, the adsorption capacity increased with increasing initial concentration, and a saturation plateau was observed when the initial DAG concentration was 1.872 mg/mL. Therefore, this concentration of DAG was selected for the following test. Table 3 lists the two model parameters. The calculated correlation coefficients of the Langmuir model were higher than those of the Freundlich model, and the correlation coefficients of the Langmuir model with HPD300 were higher than those of HPD100. This implied that the Langmuir isotherms could explain the adsorption process more suitably than the Freundlich isotherms, and that HPD300 was superior to HPD100. These results suggested that there was monolayer coverage of DAG on the resin. The theoretical maximum adsorption capacity *Q*_0_ on the HPD100 resins (HPD300) as determined from the Langmuir equation was 11.07 mg/g and 18.15 mg/g. Therefore, the HPD300 resin performed better than the HPD100 resins in both the dynamic adsorption and desorption tests.

**Table 3:** Isotherm parameters of DAG on HPD100 and HPD300 resins at 25 °C.

Resin	HPD100	HPD300
**Freundlich**		
Linear equation	ln *Q*_*e*_ = 0.2010 In *C*_*e*_ + 2.2948	ln *Q*_*e*_ = 0.2785 ln *C*_*e*_ + 2.7649
*K*_*F*_	9.9225	15.8775
1/*n*	0.2010	0.2785
*R* ^2^	0.7802	0.8883
**Langmuir**		
Linear equation	*Ce*/*Q*_*e*_ = 0.0903 *C*_*e*_ + 0.0038	*C*_*e*_/*Q*_*e*_ = 0.0551 *C*_*e*_ + 0.0033
*C* (mg/g)	11.0742	18.1488
*K*_*L*_	23.3632	16.6970
*R* ^2^	0.9991	0.9995

### Dynamic adsorption-desorption tests

**Figure 2:**
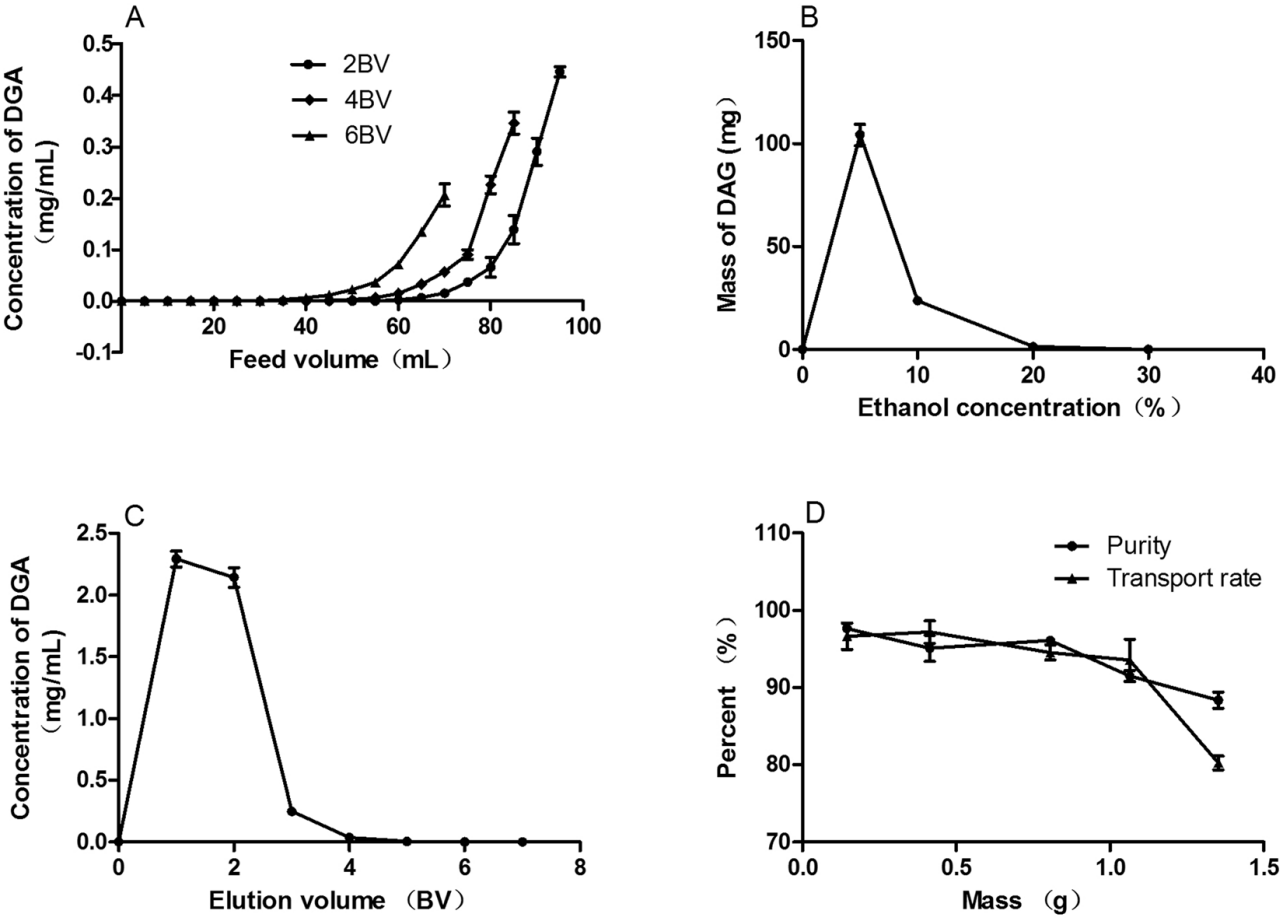
Dynamic adsorption and desorption test curves on HPD300 resin. (**A**) dynamic leakage curve; (**B**) gradient elution curve; (**C**) isocratic desorption curve and (**D**) C18 chromatographic curve of DAG on HPD300 resin column at 25 °C.

A dynamic breakthrough point was observed based on the feeding concentration, the volume and the flow rate. The initial feeding concentration of DAG was 1.87 mg/mL, and then a change in flow rate was observed at 25 °C. As shown in Fig. 2A, the different flow rates exhibited noticeably different breakthrough points. The best adsorption performance could be obtained when the flow rate was 2 BV/h. The breakthrough volume of DAG on the HPD300 resin was 75 mL (about 2.5 BV) at a flow rate of 2 BV/h, at which point the DAG concentration in the effluents reached a level that was 2% of the initial concentration^16^. The adsorption capacity of DAG on the HPD300 resin was 16.54 mg/g of dry resin.

After adsorption equilibrium was reached, the concentration and volume of eluent used in desorption were tested through gradient and isocratic elution modes at a flow rate of 2 BV/h. Figure 2B shows that DAG was fully eluted by 10% v/v ethanol aqueous solution. Therefore, a 10% v/v ethanol aqueous solution was used as the eluent in the isocratic elution process. Figure 2C shows that the concentration of DAG reached its maximum value at 1 BV of the eluent. When the eluent volume reached 4 BV, DAG was fully desorbed from the HPD300 resin. Under the above optimized conditions, 4 BV of 10% v/v elution was collected with a recovery rate of 94.17% for DAG (Table 3) and concentrated until dried in a vacuum at 60 °C to obtain a refined product.

### Purification of DAG by subsiding ethyl acetate and performing chromatography on SiliaSphere C18

To increase the purity of DAG, the product of refined using HPD300 was refined by subsiding ethyl acetate. After the ethyl acetate subsided, the purity and recovery of the refined product were 39.20% and 89.19% (Table 4), respectively. The content of DAG in the refined product that had subsided had increased 160%. The sediments were freezer the next test. To obtain purified DAG, reversed-phase chromatography was adopted due to its relatively high rate of separation and ease of scale-up. As for reversed-phase chromatography separation, Fig. 2D shows that the sample quantity of ethyl acetate subsiding into 40 g of C18 packing was 0.8 g, and the purity and recovery of DAG in the final product were 95.64% and 94.65%, respectively (Table 4).

**Table 4:** The purities and recoveries of DAG in the three-step purification.

Step	Purity (%)	Recovery (%)	Yield (mg)
Crude extract	5.06	—	—
HPD300 resin	23.69	94.17	559^a^
Subsided Ethyl acetate	39.20	89.19	4607.1^b^
SiliaSphere C18.	95.64	94.65	312.5^c^

^a^The amount of refined sample was obtained from 32.7 g of raw material by HPD300 resin.

^b^The amount of DAG was obtained from 8.55 g of refined sample of subsided ethyl acetate.

^c^The amount of DAG was obtained from 800 mg of refined sample of subsided ethyl acetate.

### Purity analysis and identification of DAG

**Figure 3:**
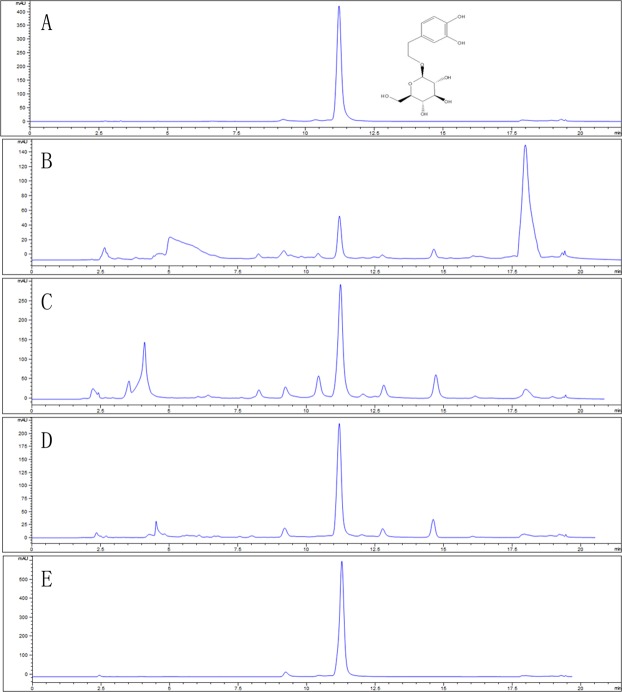
HPLC chromatograms of DAG standards (**A**); samples before treatment (**B**) and after treatment on HPD300 resin (**C**); subsided ethyl acetate of DAG (**D**) and C18 chromatography of DAG (**E**).

After the three-step purification, the purity of DAG increased from 5.06% of *S. cuneata* extracts to 23.69% of resin purity; then, it increased to 39.20% of the subsided ethyl acetate. Finally, it increased to 95.64% of reversed-phase chromatography. The total recovery rate was 79.50%. The HPLC chromatograms created through three-step purification are compared in Fig. 3. Through an NMR analysis, the chemical structure of DAG was identified, and the data were as follows: ^1^H-NMR (DMSO-d_6_, 400 MHz) δ: 6.68 (1H, d, J = 2 Hz, H-2), 6.66 (1H, d, J = 8.4 Hz, H-5), 6.54 (1H, dd, J = 1.6 and 8.0 Hz, H-6), 4.28 (1H, d, J = 8.0 Hz, H-1′), 2.81–4.01 (6H, m, Ha-2′,3′,4′,5′,6′); ^13^C-NMR (DMSO-d_6_, 100 MHz) δc: 146.1 (C-3), 144.6 (C-4), 131.5 (C-1), 121.2 (C-6), 117.1 (C-5), 116.3 (C-2), 104.4 (C-1′), 71.6 (C-8), 36.6 (C-7), 78.1 (C-5′), 77.9 (C-3′′), 75.1 (C-2′), 72.1 (C-4′), 62.7 (C-6′). The data were identical to those reported in literature^17^.

### DAG protected mice against DSS-induced acute colitis

**Figure 4:**
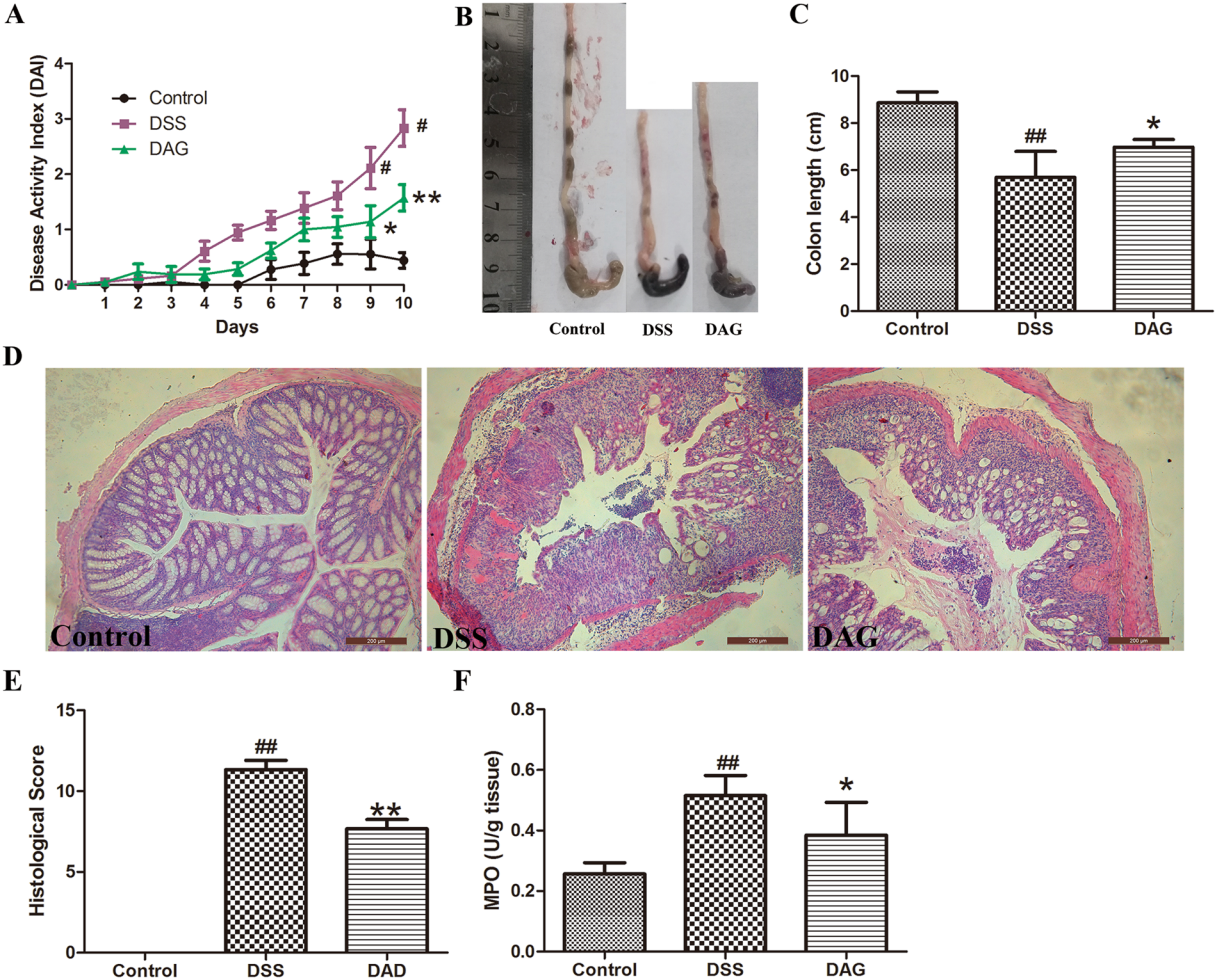
DAG protected mice against DSS- induced acute colitis. (**A**) The disease activity index changes were detected daily. (**B** and **C**) Colon length was measured after 7 d of DSS administration. (**D** and **E**) Colon tissue sections stained with hematoxylin and eosin. (**F**) Effects of DAG on the MPO activity of colonic tissue. The values presented are the mean ± S.E.M (n = 8 in each group). ^##^*P* < 0.01 compared with the control group, **P < 0.01 compared with the DSS group, *P < 0.05 compared with the DSS group.

DAG was isolated from the stem of *S. cuneata*, which is a well-known traditional Chinese medicine for treating ulcers. Because hydroxytyrosol, the metabolite of DAG, has significantly ameliorated colitis in animal experiments, we investigated the pharmacological effects of DAG on DSS-induced acute colitis. We established a colitis mode with 3% DSS for 7 d. The disease activity index (DAI) was evaluated as previously published^18^. As shown in Fig. 4A, the DAI scores markedly increased after DSS administration compared with those of the control group. Moreover, DAG pretreatment significantly decreased the DAI. In addition, as shown in Fig. 4B,C, the colon length of the DSS group shortened more than that of the control group. DAG pretreatment significantly reversed the shortening of colon length. We further evaluated the effects of DAG pretreatment on the histopathology and MPO activity. As shown in Fig. 4D,E, DSS-induced colon pathological changes were markedly reversed by DAG pretreatment. Moreover, as shown in Fig. 4F, the MPO activity, a marker for neutrophil burden, was significantly increased by DSS treatment. However, this change was significantly reduced through DAG pretreatment.

### DAG ameliorated intestinal inflammation and increased antioxidant enzyme activities in colitis

**Figure 5:**
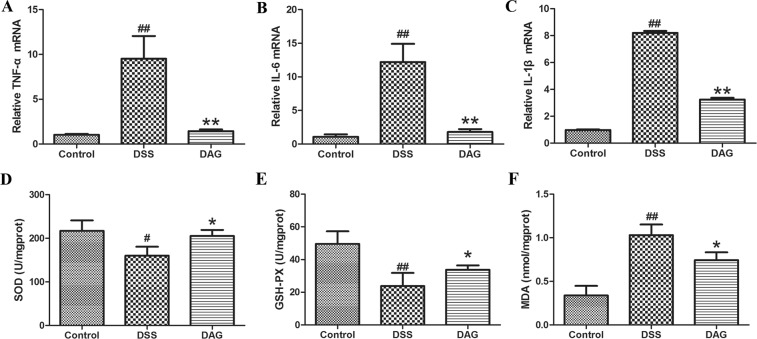
DAE reduced the mRNA levels of pro-inflammatory cytokines IL-6 (**A**), TNF-α (**B**), and IL-1β (**C**) as analyzed through qRT-PCR. DAE reduced oxidative stress in the colon. (**D**) SOD activity (**E**), GPx activity and (**F**) MDA levels. Data are expressed as means ± SEM (n = 8 per group). ^##^*P* < 0.01 compared with the control group, ^#^*P* < 0.05 compared with the control group, **P < 0.01 compared with the DSS group, *P < 0.05 compared with the DSS group.

A growing body of evidence demonstrates that the overproduction of pro-inflammatory cytokines and oxidative stress plays a critical role in the pathogenesis of ulcerative colitis. Therefore, we studied the effects of DAG on the mRNA levels of major pro-inflammatory cytokines, the activity of antioxidant enzymes and the levels of malondialdehyde (MDA). As shown in Fig. 5A–C, the mRNA levels of TNF-a, IL-6 and IL-1β markedly increased after DSS administration compared with those of saline-treated controls. TNF-a, IL-6 and IL-1β mRNA were significantly attenuated by DAG pretreatment compared with those of the DSS group. As shown in Fig. 5D and E, the activities of antioxidant enzymes SOD and GPx evidently decreased after DSS administration compared with those of saline-treated controls. In contrast, DAG pretreatment significantly increased the activities of SOD and GPx. Additionally, as shown in Fig. 5F, we found that DSS-induced elevated MDA was significantly counteracted by DAG pretreatment.

### DAG suppressed ROS and proinflammatory cytokine production in LPS-stimulated RAW 264.7 macrophages

**Figure 6:**
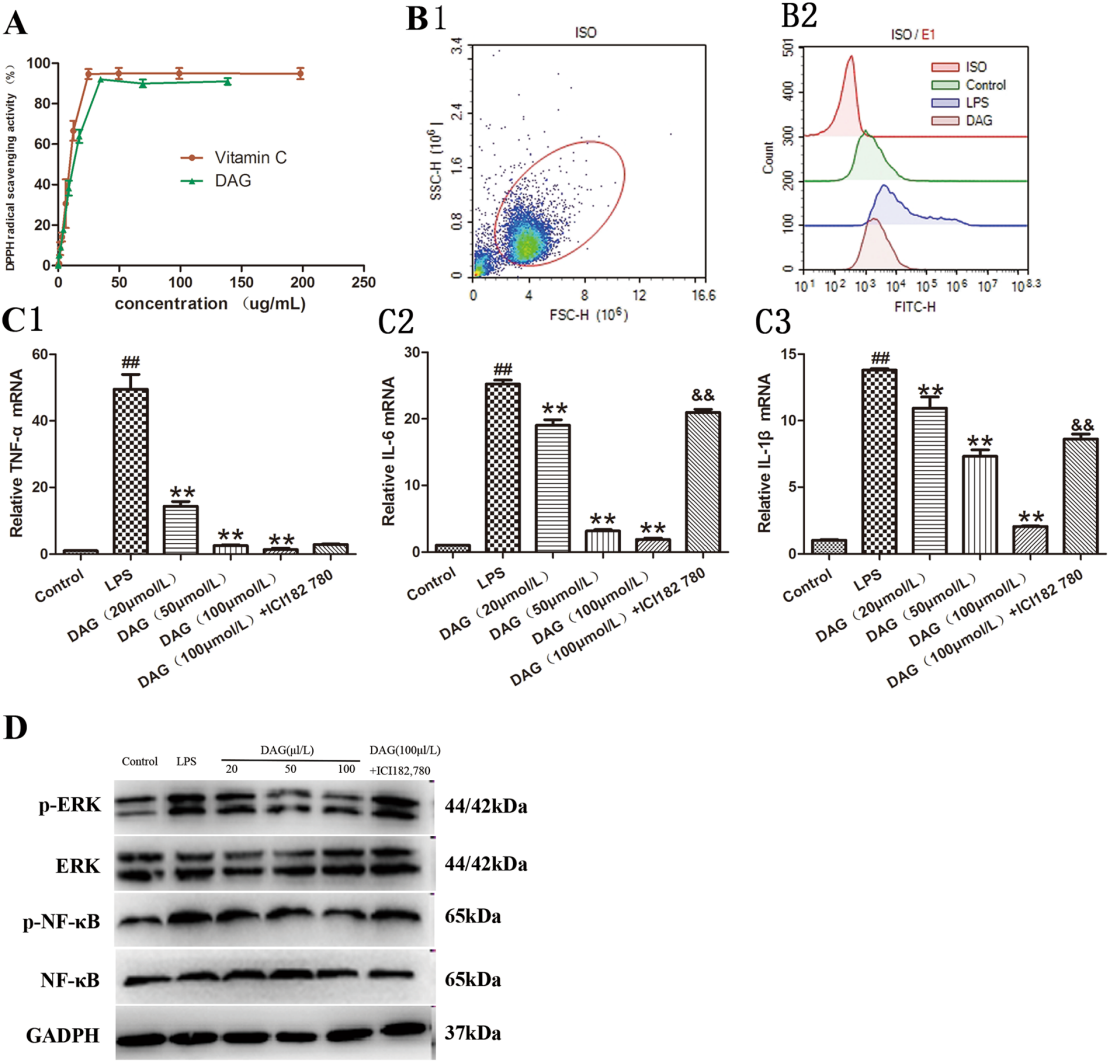
(**A**) DPPH scavenging activity (**B**) ROS level in RAW 264.7 cells was detected by DCFH-DA staining (**C**) mRNA expression levels of TNF-α, IL-1β, and IL-6 (n = 4). (**D**) NF-κB/P-NF-κB and ERK1/2/P-ERK1/2 protein levels after ER antagonist, ICI 182,780, treatment in RAW 264.7 macrophages.

To further evaluate the antioxidant and anti-inflammatory activities *in vitro*, we first evaluated the DPPH scavenging activity. As shown in Fig. 6A, the IC50 values of DAG and Vc were 17.03 and 12.07 ug/mL, respectively. Additionally, as shown in Fig. 6B1,B2, LPS caused the overproduction of ROS in RAW 264.7 macrophages, while DAG pretreatment significantly decreased the production of ROS in a concentration-dependent manner. To further explore the possible mechanisms underlying the anti-inflammatory effects of DAG on LPS-stimulated RAW264.7 cell inflammation, the pure ER antagonist, ICI 182,780, was used. As shown in Fig. 6C1–C3, the TNF-a, IL-6 and IL-1β mRNA markedly increased in RAW 264.7 cells after LPS exposure, whereas those increases were significantly attenuated by DAG pretreatment in a concentration-dependent manner, and ICI 182,780 reversed the DAG-mediated effects. Because NF-κB and ERK1/2 are 2 key pathways for LPS-stimulated signaling events, we measured the activation of the ERK1/2 and NF-κB inflammatory signaling pathways. As shown in Fig. 6D, the increased phosphorylation of ERK1/2 and NF-κB was observed in LPS-stimulated RAW264.7 cells. DAG pretreatment significantly decreased the phosphorylation of ERK1/2, and NF-κB and ICI 182,780 reversed the DAG-mediated effects.

## Discussion

### Isolation and purification of DAG from the stem of *S. cuneata*

In this study, DAG purification methods were established through comparisons, and then the processing conditions were optimized to yield highly pure DAG from the stem of *S. cuneata*. Macroporous resin has unique adsorption and screening properties with a high adsorption capacity and selectivity, good stability, fast adsorption and desorption under mild conditions, ease of regeneration and cost savings. It has been widely used for the enrichment and purification of the natural products of PhGs^11,12^. During phase I of this study, the HPD300 macroporous resin was selected for the isolation and purification of DAG through static adsorption and desorption tests. In the dynamic adsorption and desorption tests, the flow rate of the loaded sample and the concentration and volume of the eluent used in desorption were found to be optimal. The DAG purity increased by 5 times.

### Protective effects of *S. cuneata*-derived DAG against DSS-induced colitis in mice

DAG is one of the phenylethanoid glycosides found in many medicinal plants; however, its pharmacological effects have not been investigated. In this study, we investigated the antioxidant and anti-inflammatory activities of DAG *in vivo* and vitro. Clinical and experimental studies have shown that uncontrolled inflammatory responses and oxidative stress damage play crucial roles in the pathophysiology of colitis^19,20^. In this study, we designed a series of experiments to clarify whether the antioxidant and anti-inflammatory activities of DAG contributed to the amelioration of DSS-induced acute colitis in mice. We observed that DAG treatment significantly relieved colitis symptoms, such as shortening of colon length and an increase in DAI scores and colonic tissue damage. Moreover, oxidative stress and cytokine expression significantly decreased in the colon. Evidence demonstrates that the down-regulation of the inflammatory responses could ameliorate UC^21^. Our data clearly demonstrated that DAG pretreatment significantly inhibited DSS-induced high production of pro-inflammatory cytokines. Furthermore, DAG decreased the mRNA expression of pro-inflammatory cytokines and suppressed the ERK1/2 and NF-κB signaling pathways in LPS-induced RAW 264.7 macrophages. In addition, MPO is known to be involved in the production of oxidative stress that is involved in the pathogenesis of ulcerative colitis^22^. DAG pretreatment caused a marked decrease in MPO activities induced by DSS exposure. It also decreased MDA levels and increased superoxide dismutases (SOD) and glutathione peroxidase (GPx) activity in colonic tissues. Furthermore, DAG demonstrated free radical scavenging activity using the DPPH. Additionally, DAG suppressed the levels of ROS in LPS-stimulated RAW 264.7 macrophages. These results suggested that the protective effects of DAG could be realized by inhibiting the generation of pro-inflammatory cytokines and oxidative stress in DSS-induced colitis. DAG can be converted to hydroxytyrosol after oral administration by intestinal bacteria, and several studies have indicated that hydroxytyrosol can reduce cancer growth and induce cell cycle arrest and apoptosis in colon cancer^23,24^. Thus, it is possible that DAG can also act as anticancer agent; however, this remains to be determined.

In summary, this study proved that DAG can be generated using biological extraction and plays an extensive role in antioxidant and anti-inflammatory activities. Consequently, the study generated an important scientific discovery for those engaged in drug discovery. More studies are necessary to further understand the pharmacological effects of DAG.

## Materials and Methods

### Adsorbents, reagents and preparation of samples

Macroporous resins including AB-8, X-5 and KNA-9 were purchased from Nankai Hecheng S&T Co., Ltd (Tianjin, China). HPD100, HPD300, HPD5000, HPD450, HPD750 and DM130 were obtained from Cangzhou Bon Adsorber Technology Co., Ltd. (Hebei, China). Table S1 shows their physical properties. Before use, these resins were soaked in 95% v/v ethanol aqueous solutions for 24 h, washed with a 4% HCl aqueous solution, deionized water, 4% NaOH aqueous solution and finally washed with deionized water until a pH of 7.0 was reached. SiliaSphere C18 was purchased from Greenherbs S&T Co., Ltd. (Beijing, China).

HPLC-grade acetonitrile was bought from Concord Technology Co., Ltd (Tianjin, China). 1,1-diphenyl-2-picrylhydrazyl (DPPH) was obtained from sigma Chemical Co. (Sigma-Aldrich GmbH, Sternheim, Germany). Other reagents and chemicals were analytical grade. MPO, SOD, GPx and MDA Detection Kits were purchased from Nanjing Jiancheng Technology Co. Ltd. (Nanjing, China). Trizol reagent was purchased from Takara Bio Inc. (Shiga, Japan).

Ground *S. cuneata* stem (1.0 kg) was extracted with 95% v/v ethanol aqueous solutions (1:10, sample: water, w/w) under reflux for 60 min, and this process was then repeated twice. The extracted solutions were combined and filtered, and the filtrate was concentrated to a volume of 400 mL by a rotary evaporator under a vacuum at 60 °C. Then, 400 mL of residue was added to a 2% gelatin solution to subside for 24 h. It was then filtered, and the filtrate was concentrated to dryness by a rotary evaporator under a vacuum at 60 °C. The residue was dissolved in deionized water to be used as stock solution for the subsequent test.

### HPLC analysis of DAG

An Agilent 1260 infinity HPLC system was used (Agilent, Agilent Technologies. Inc., USA). An ODS-2 Hypersil C-18 column (250 mm × 4.6 mm id, 5 μm; Thermo Scientific, Thermo Fisher Scientific Inc., USA) was used at a column temperature of 35 °C. The mobile phase was acetonitrile (A) and water (B). The elution system was performed using a stepwise gradient elution of 0–14 min, 95–87% B; 14–15 min, 87–85% B; 15–16 min, 85–95% B; 16–21 min, 95–95% B. The flow rate was set at 0.9 mL/min. The effluent was monitored at 278 nm. The standard solution of DAG was determined. It showed a good linearity over a range of 0.4–16 μg for DAG. The regression equation for DAG was Y = 420.882012X + 122.39368, (R^2^ = 0.9995, n = 6), where Y is the peak area and X is the mass.

### Static adsorption and desorption tests

Adsorption resin screening was conducted. Pretreated resin (equivalent to 0.5 g dry resin) was added to a 10-mL sample solution. After the mixed solutions were shaken (180 rpm) for 24 h, the resins were washed in deionized water and desorbed with 10 mL 95% v/v ethanol. The solutions after adsorption and desorption were analyzed through HPLC. The following equations were used to evaluate the adsorption capacity, adsorption ratio and desorption ratio:

Adsorption capacity:

1 $${Q}_{e}=\frac{{V}_{i}({C}_{0}-{C}_{e})}{W}$$

Adsorption ratio:

2 $$E=\frac{{C}_{0}-{C}_{e}}{{C}_{0}}\times 100 \% ,$$

where *Q*_*e*_ is the adsorption capacity at the adsorption equilibrium (mg/g dry resin); *E* is the adsorption ratio (%); *V*_*i*_ is the initial volume of feed solution (mL); *C*_0_ and *C*_*e*_ are the initial and equilibrium concentrations of DAG in solutions, respectively (mg/mL); and *W* is the weight of the dry resin (g).

Desorption ratio:

3 $$D=\frac{{C}_{d}{V}_{d}}{{V}_{i}({C}_{0}-{C}_{e})}\times 100 \% ,$$

where *D* is the desorption ratio (%); *C*_*d*_ is the concentration of liriodendrin in the desorption solution (mg/mL); *V*_*d*_ is the volume of the desorption solution (mL); and *V*_*i*_, *C*_0_ and *C*_*e*_ are the same as defined above.

The adsorption kinetics were analyzed. The adsorption kinetic of resins was studied by mixing 10 mL of sample solution and pretreated resin. The DAG concentration in the solution phase was analyzed through HPLC at different time intervals. To illustrate the underlying mechanism of the adsorption kinetic process, three models were adopted to describe the adsorption process^25^.

The equation of the pseudo-first-order kinetic model was as follows:

4 $$\mathrm{ln}({Q}_{e}-{Q}_{t})=\,\mathrm{ln}\,{Q}_{e}-{k}_{1}t$$

The equation of the pseudo-second-order kinetic model was as follows:

5 $$\frac{t}{{Q}_{t}}=\frac{t}{{Q}_{e}}+\frac{1}{{{Q}_{e}}^{2}{k}_{2}}$$

The equation of the intra-particle diffusion kinetic model was as follows:

6 $${Q}_{t}={k}_{i}{t}^{0.5}+C,$$

Where *Q*_*e*_ and *Q*_*t*_ are the adsorption capacity at equilibrium and at any time *t* (mg/g dry resin), respectively; *k*_1_ (min^−1^), *k*_2_ ($g/mg\cdot \,\min$) and *k*_*i*_ ($mg/g\cdot {\min }^{0.5}$) are the rate constants of the above three models; *c* is the constant, representing the boundary layer thickness (mg/g).

Adsorption isotherms were studied. The equilibrium adsorption isotherms of DAG on resins were studied through the following experiment. Pretreated resin and 10 mL of sample solutions of different concentrations were mixed. The equilibrium concentration of DAG was analyzed. Freundlich and Langmuir equations were used to describe the adsorption behavior^26,27^.

The Freundlich equation was as follows:

7 $${Q}_{e}={K}_{F}{C}_{e}^{1/n}$$

The Langmuir equation was as follows:

8 $${Q}_{e}=\frac{{Q}_{0}{K}_{L}{C}_{e}}{1+{K}_{L}{C}_{e}},$$

where *K*_*F*_ is the Freundlich constant, an indicator of adsorption capacity; 1/*n* is an empirical constant related to the magnitude of the adsorption driving force; *Q*_0_ is the theoretical maximum adsorption capacity (mg/g dry resin); *K*_*L*_ is the Langmuir adsorption equilibrium constant; and *Q*_*e*_ and *C*_*e*_ are the same parameters used in equation (1).

### Dynamic adsorption and desorption tests

Dynamic adsorption/desorption experiments were performed in a glass column wet-packed with resin. For the dynamic breakthrough experiment, a sample solution was loaded into the pretreated glass column at a 2, 4 and 6 BV/h flow rate. The DAG concentration in the effluents that were collected at 5-mL intervals was analyzed. After adsorption equilibrium, desorption was conducted with the percentage of ethanol (0, 5, 10, 20, 30, 40, 50, 60, 80 and 95% v/v) successively at a flow rate 1 BV/h. The volume of each ethanol elution was 3 BV, and the concentration of DAG in each effluent was determined through HPLC. For the quantity of ethanol elution, the experiment was performed as follows when the adsorption equilibrium was reached: the resin column was washed first with 3 BV deionized water and was then eluted with 10% v/v ethanol 6 BV at a flow rate of 1 BV/h. Every 1 BV of elution was collected and monitored through HPLC.

### Subsiding of ethyl acetate and purification through chromatography on SiliaSphere C18

The 10% v/v ethanol elution was evaporated until dry and then extracted three times with methanol in an ultrasonic bath. The filtrate was concentrated to a fixed volume. Then, a five-fold volume of ethyl acetate was added to the concentrate liquid and vigorously stirred. The sediments were then freeze-dried.

SiliaSphere C18 (40 g) was packed in a glass column. The column was pre-equilibrated with 8% v/v acetonitrile. The refined product of subsided ethyl acetate was dissolved in 8% v/v acetonitrile to obtain solutions of different concentrations and to then load them onto the column for pre-equilibration. The elution process was conducted with the 8% v/v acetonitrile at a low pressure, and every 10 mL of elution was collected and monitored through thin layer chromatography (TLC) (Ethyl acetate: methanol = 4:1 as the developing solvent, 10% sulfuric acid ethanol solution as the chromogenic agent). Then, according to spots of DAG as shown in TLC, the elution solutions were collected and analyzed.

### Antioxidant activity

An assay of DPPH radical scavenging was evaluated. The scavenging effect of the DPPH radical was evaluated using the method reported^28^. Serial dilutions of the DAG in methanol (2 mL) were incubated with a 2-mL methanol solution of DPPH (0.1 mM/L) for 30 min. The adsorption was recorded at 517 nm against a control at room temperature. Vc was used as a positive control. The DPPH radical scavenging activity was calculated using the following equation:

9 $$DPP{H}^{\bullet }\,scavenging\,effect\,( \% )=\frac{{A}_{control}-{A}_{sample}}{{A}_{control}}\times 100$$

### Animals and experimental designs

SPF male BALB/c mice (25 ± 2 g) were purchased from Beijing Vital River Laboratory Animal Technology Co. Ltd. (Beijing, China). All animal experiments were performed strictly under the guidelines on laboratory animals of and the animal protocols were approved by the Ethics Committee at the Tianjin Medical University (Permit No: TMUaMEC 2018028). Mice were given at least one week to adjust to the environment (i.e., an experimental room with 12 h of light/dark) before use. The mice were randomly divided into three groups (n = 8 each): a control group, a DSS group and DAG plus DSS group. Acute colitis was induced in each group through the administration of 3% (w/v) DSS in drinking water for a week. DAG (20 mg/kg) was orally pre-administered for 3 d before DSS treatment, and each group was treated for one week with DSS exposure. After 7 d of DSS ingestion, all mice were sacrificed, and the colons from the ileocecal junction to the anal verge were removed, rinsed with cold PBS, and then the lengths of the colons were measured.

### Cell culture

Cells of the murine macrophage-like RAW264.7 cell line were obtained from the American Type Culture Collection (Manassas, VA, USA). Cells were cultured in an RPMI 1640 medium supplemented with 100 U/ml penicillin, 100 μg/mL streptomycin and 10% (v/v) 10% fetal bovine serum and were then maintained at 37 °C in a humidified atmosphere of 5% CO_2_. Cells were seeded on plates before being subjected to treatments. DEA was added 2 h before LPS stimulation.

### Quantitative Real-Time Polymerase Chain Reaction

Total RNA was extracted from the colons or RAW264.7 cells using Trizol reagent, and complementary DNA transcription was performed using a TransGen Reverse Transcription Kit according to the manufacturer’s protocol. A quantitative real-time polymerase chain reaction (qRT-PCR) was performed using SYBR Green Master Mix (Promega). Oligonucleotide primers were shown in Table S2.

### MPO, SOD, MDA and GPx

MPO activity, an indicator of polymorphonuclear leukocyte accumulation, was measured with an MPO Detection Kit. The levels of SOD, GPx and MDA were all determined through commercial kits according to the manufacturer’s instructions.

### Histologic examination and grading

For histopathological assessment, the distal colons were fixed in 10% buffered formalin, embedded in paraffin and then cut into 5-μm sections for hematoxylin-eosin (H&E) staining. The mean pathological index was evaluated as previously described^14^.

### Western Blot analysis

Total proteins were extracted using cold RIPA (Millipore) containing a protease inhibitor. The protein concentration was centrifuged to remove the pellet and debris. Total proteins were separated by 10% SDS-PAGE, transferred to polyvinylidene fluoride (PVDF) membranes (Millipore) and then blocked with 5% fat-free milk. Expressions of NF-κB, P- NF-κB, ERK1/2, and P-ERK1/2 (Cell Signaling) and GAPDH (Abcam) proteins were determined using the respective specific antibodies (1/1000). After a membrane was incubated with a secondary antibody, it was then visualized using an enhanced chemiluminescence system (Bio-Rad).

### Statistical analysis

All the results were expressed as the mean ± standard deviation (*n* = 3), and the data were analyzed using one-way ANOVA (analysis of variance) using SPSS PC version 17. Statistical significance was determined at a P value < 0.05.

## Supplementary information

sopplementary datatable
