# Supplementary material for: Purification of 3, 4-dihydroxyphenylethyl alcohol glycoside from Sargentodoxa cuneata (Oliv.) Rehd. et Wils. and its protective effects against DSS-induced colitis
Source: Sci Rep. 2019 Mar 1;9:3222. doi: 10.1038/s41598-019-38926-8 (PMC6397144; doi:10.1038/s41598-019-38926-8)
Supplement: Supplementary file 1 — sopplementary datatable [file 41598_2019_38926_MOESM1_ESM.pdf]

Purification of 3, 4-dihydroxyphenylethyl alcohol glycoside from *Sargentodoxa cuneat*a (Oliv.) Rehd. et Wils. and its protective effects against DSS-induced colitis

Dihua Li[1], Yuzhen Zhuo[1], Qi Zhang[1], Lanqiu Zhang[1], Shukun Zhang[1], Yuanshan Lv[1], Caixia Li[1], Lihua Cui[1], Xin Guan[1], Lei Yang[1, ¶], Ximo Wang[1, 2, ¶]

Table S1 Physical properties of nine macroporous resins in testing.

Resin	Average pore diameter (nm)	Surface area (m^2/g)	Polarity
AB-8	13−14	450−530	Non-polar
X-5	29−30	500−600	Non-polar
HPD100	8.5−9	650−700	Non-polar
HPD300	5-5.5	800-870	Non-polar
HPD5000	10-11	⩾400	Non-polar
HPD450	9-11	500−550	Middle-polar

HPD750	8.5-9	650-700	Middle-polar
DM130	9-10	500-550	Middle-polar
NKA-9	15.5-16.5	170-250	Polar

Table S2 Forward primer and reverse primer

	Forward primer	Reverse primer
TNF-a	5′- CGTCAGCCGATTTGCTATCT-3′	5′-CGGACTCCGCAAAGTCTAAG-3′
IL-6	5′-AGTTGCCTTCTTGGGACTGA-3′	5′- TCCACGATTTCCCAGAGAAC-3′
IL-1β	5′-CTATGTCTTGCCCGTGGAG-3′	5′- CATCATCCCACGAGTCACA-3′
GADPH	5′-GCCTCGTCTCATAGACAAGATG-3′	5′- CAGTAGACTCCACGACATAC-3′
